# Supplementary material for: Downregulation of Serotonergic System Components in an Experimentally Induced Cryptorchidism in Rabbits
Source: Int J Mol Sci. 2024 Mar 9;25(6):3149. doi: 10.3390/ijms25063149 (PMC10970345; doi:10.3390/ijms25063149)
Supplement: Supplementary file 1 [file ijms-25-03149-s001.zip › ijms-2740146-supplementary.pdf]

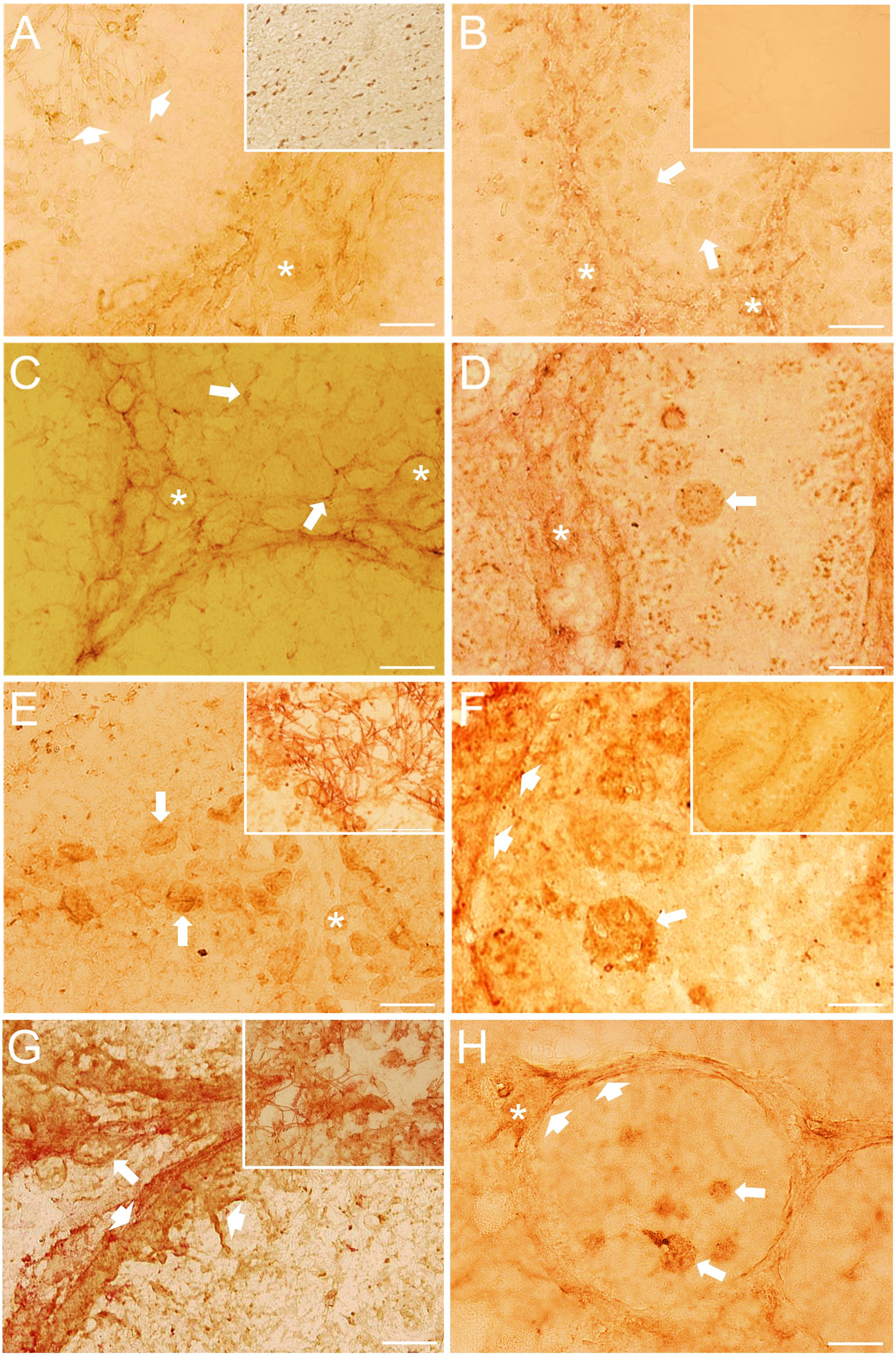
A
B
C
D
E
F
G
H

Supplementary Figure S1: Immunohistochemistry of serotonergic system elements in 150 days old control and COI rabbit testes sections (I). In control rabbits (A), TPH1 immunoreactivity was found faintly stained in interstitial zone (asterisk) and also in sperm located in the lumen of seminiferous tubules (arrowheads), inset shows brain stem neurons positive to TPH1; in COI rabbits (B), TPH1 was found mainly expressed in interstitial zone (asterisks), and in gonocyte-like cells (arrows), inset shows a negative control; in control rabbits (C), 5-HT_{1B} receptor was found expressed in interstitial zone (asterisks), and faintly stained in apparent cytoplasmic prolongations of Sertoli cells into the of seminiferous tubules (arrows), while in COI rabbits (D), it was found expressed in the interstitial zone (asterisk), and gonocyte-like cells (arrow); in control rabbits (E), 5-HT_{2A} was found expressed in spermatogonial stem cell (arrows), slightly stained in Leydig cells (asterisk) and in sperm (inset); in COI rabbits (F), it was found expressed in the interstitium, in some cytoplasmic projections which ran parallel to the basal membrane (arrowheads) and in gonocyte-like cells (arrow), inset shows a panoramic view; in control rabbits (G), 5-HT_{3A} receptor was observed strongly stained in clusters of Leydig cells in the interstitial zone (arrow), in apparent cytoplasmic prolongations of Sertoli cells next to the basal membrane (arrowheads) and in sperm (inset); in sharp contrast, in COI rabbits (H), it was found faintly stained in the interstitial region (asterisk), in regionalized zones of the basal membrane (arrowheads) and in gonocyte-like cells (arrows). Scale bar A-H: 10μm.

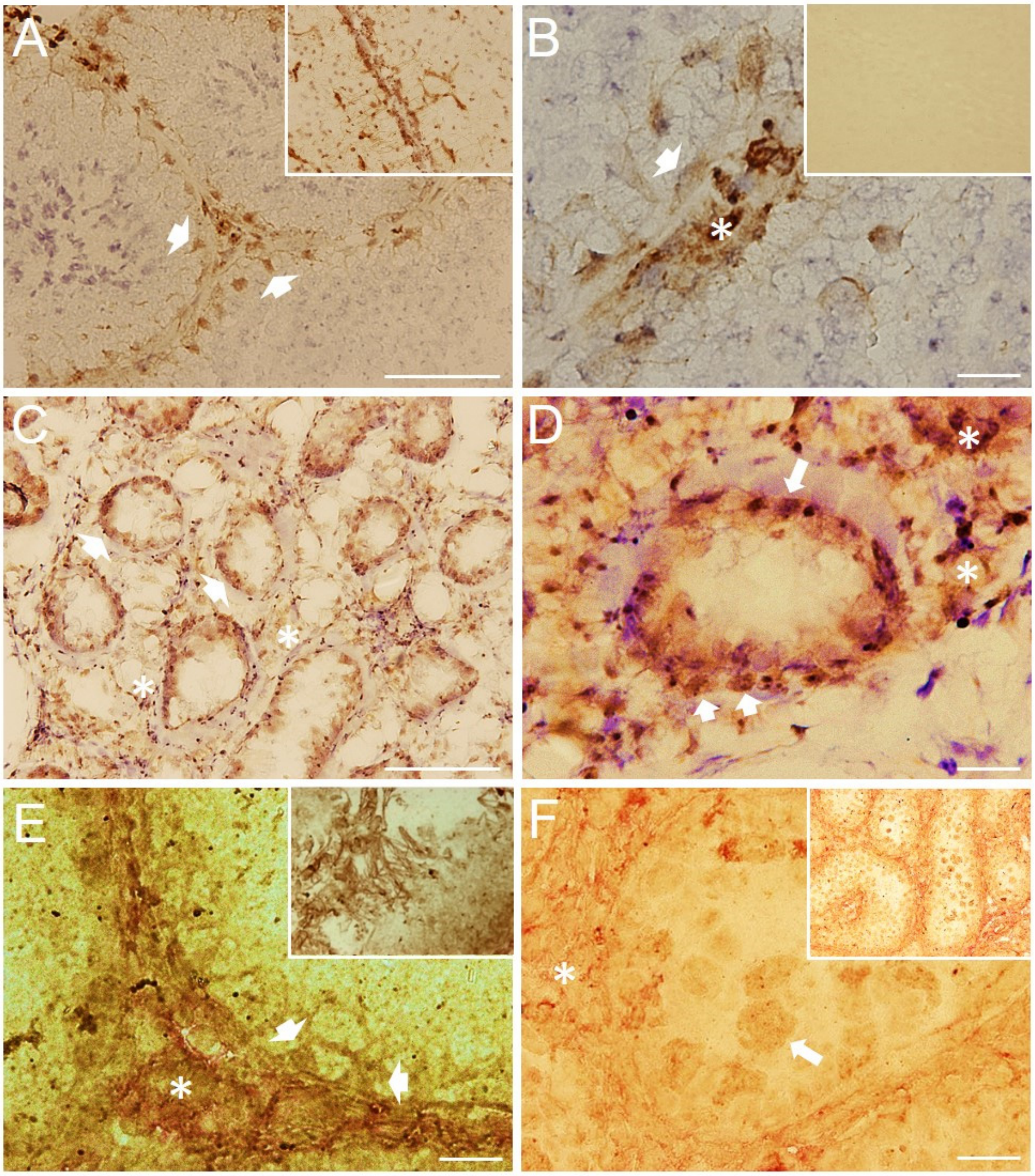

Supplementary Figure S2: Immunohistochemistry of serotonergic system elements in 150 days old control and COI rabbit testes sections (II). In control rabbits (A and B), VMAT1 was detected in cells located next to the basal membrane (A), presumptively in Sertoli cells (arrowheads), inset shows brain stem neurons used as positive control; a higher magnification is shown in (B), in which immunoreactivity is better observed with strong staining was detected in the interstitial zone, in Leydig cells (star), and Sertoli cell (arrowhead), inset shows negative control. A panoramic view of VMAT1 in COI rabbits is depicted in (C), in which a strong staining was

observed in the interstitial zone, probably in Leydig cells (stars) and also in the basal membrane of seminiferous tubules, probably in peritubular myoid and/or Sertoli cells (arrowheads); a higher magnification is shown in (D), in which positive signal in Sertoli cell-like (arrowheads), and spermatogonial stem cell (arrow) are better resolved, also Leydig cells are identified in the interstitial zone (asterisks). In control rabbits (E), 5-HT_T was strongly immunostained in the interstitial zone (asterisk), in apparent Sertoli cells (arrowheads), and sperm (inset); in sharp contrast, 5-HT_T was found faintly immunostained in the interstitial zone (asterisk) and gonocytes (arrow), inset shows a panoramic view of this transporter. Scale bar in A 50 μm and C: 100μm, in B, D, E and F: 10μm.
